# Reconstruction of Chronic Achilles Tendon Ruptures with Hamstring Autografts: Plantar Flexor Strength Is Preserved Despite Shortening of the Moment Arm

**DOI:** 10.3390/jcm15052009

**Published:** 2026-03-05

**Authors:** Bartosz Kiedrowski, Jakub Kaszyński, Karol Szapel, Paweł Bąkowski, Artur Banach, Tomasz Piontek

**Affiliations:** 1 Rehasport Clinic, ul. Górecka 30, 60-120 Poznan, Poland; jakub.kaszynski@rehasport.pl (J.K.); tomasz.piontek@rehasport.pl (T.P.); 2 Department of Spine Disorders and Pediatric Orthopedics, Poznan University of Medical Sciences, 61-545 Poznan, Poland; 3 Reumatika, Aleja Wilanowska 333, 02-665 Warsaw, Poland; 4 MicroBioRobotic Systems Laboratory, Institute of Mechanical Engineering, École Polytechnique Fédérale de Lausanne (EPFL), 1015 Lausanne, Switzerland

**Keywords:** Achilles tendon rupture, tendon reconstruction, moment arm shortening, radiographic measurement, rehabilitation, plantar flexor strength

## Abstract

**Background**: Chronic Achilles tendon ruptures present a major surgical challenge due to tendon retraction, degeneration, and large defects. Autologous hamstring tendon grafts have emerged as a reliable reconstructive option, yet their biomechanical consequences remain poorly understood. This study investigated whether Achilles tendon reconstruction with semitendinosus and gracilis autografts alters the plantar flexor moment arm and whether such changes affect muscle strength. **Methods**: A cohort of 25 patients (mean age: 44.5 years) underwent minimally invasive endoscopic reconstruction using hamstring autografts. This secondary salvage procedure was performed in patients with neglected ruptures or failed primary treatment. Five patients were excluded from the original intervention group due to inadequate radiographic quality. Radiographic measurements of the Achilles tendon moment arm and isometric plantar flexor strength assessments were performed at 12 and 24 months postoperatively. Statistical analyses included paired *t*-tests, Wilcoxon signed-rank tests, and correlation analyses. **Results**: Results showed a significant shortening of the Achilles tendon moment arm after reconstruction compared with the preoperative imaging length (mean reduction: 6.6 mm; *p* < 0.0001). Despite this, plantar flexor strength in the operated limb improved significantly over time at 12 and 24 months (+388.6 N at 24 months; *p* = 0.0067) and did not correlate with the degree of moment arm shortening (*p* > 0.3). By 24 months, the operated limb demonstrated comparable or greater strength than the contralateral side, with nearly half of the patients achieving substantial clinically meaningful improvements. **Conclusions**: In conclusion, Achilles tendon reconstruction with hamstring autografts leads to consistent moment arm shortening, yet this does not impair long-term restoration of plantar flexor strength. A progressive rehabilitation program extending up to two years appears essential to optimize recovery and compensate for biomechanical alterations.

## 1. Introduction

Chronic Achilles tendon lesions, often resulting from neglected ruptures, failed repairs, or advanced tendinopathy, pose a significant challenge due to tendon retraction, degeneration, and substantial defects exceeding 3 cm [1,2,3]. In a 10-year population-based cohort study, Raikin et al. reported that 24% (96/406) of Achilles tendon ruptures were classified as chronic at presentation (>4 weeks from injury), whereas 76% were acute (<4 weeks), although a discrete population-level annual incidence for chronic or neglected Achilles tendon ruptures has not been independently established [4]. While primary repairs such as open and percutaneous suture are effective in acute injuries, chronic cases frequently require tendon reconstruction with graft augmentation to restore functional integrity and prevent recurrence [5,6,7]. In the literature, various reconstructive surgical techniques have been described, including tendon transfers using the flexor hallucis longus, peroneus brevis, hamstring tendons, and gastrocnemius–soleus turndown flaps [8,9,10]. Although these methods generally yield comparable functional outcomes, there are no established guidelines regarding the optimal choice of technique [8]. Among the available reconstructive options for chronic Achilles tendon ruptures, hamstring tendon transfer has been reported to be associated with relatively low complication rates and favorable long-term clinical outcomes [9,10]. A surgical technique utilizing semitendinosus and gracilis tendon autografts for chronic Achilles tendon lesions was previously developed and reported [11]. This technique should be considered a salvage reconstruction strategy rather than a first-line procedure, as it is specifically indicated for chronic and neglected Achilles tendon ruptures and chronic partial tears in which primary end-to-end repair is not feasible due to tendon retraction, compromised tissue quality, or substantial gap formation necessitating reconstructive grafting [11]. The minimally invasive endoscopic technique used in this study was developed to reduce soft tissue damage, provide stable cortical fixation with controlled graft tension, and allow for more precise restoration of the Achilles tendon length–tension relationship in cases where primary repair is not feasible. However, as with all reconstructive procedures, potential drawbacks include donor site complications, wound healing complications, graft elongation, infection, and incomplete recovery of donor site strength. 

Despite advances in surgical and rehabilitation techniques, Achilles tendon reconstruction carries the risk of biomechanical alterations, including shortening of the triceps surae muscle moment arm [4]. The moment arm, defined as the perpendicular distance between the ankle joint’s axis of rotation and the muscle’s line of action, is a critical determinant of torque production at the joint [6]. Shortening of the moment arm, resulting from displacement of tendon attachment sites relative to the native insertion, alterations in tendon elasticity, or thickening at the reconstruction site, may compromise the functional strength of the plantar flexor muscles, thereby impairing patients’ ability to perform daily activities and high-demand athletic tasks [12].

Since 1990, numerous studies have estimated the Achilles tendon moment arm using various imaging modalities, including radiography, ultrasonography, and magnetic resonance imaging [13,14,15,16,17,18,19,20]. However, the majority of existing investigations have been conducted either on healthy participants or cadaveric models. The only available postoperative data pertain to patients who underwent total ankle arthroplasty [20]. To date, no prior study has assessed changes in the Achilles tendon moment arm following surgical reconstruction with graft augmentation.

This study is based on the same patient cohort previously reported in our 2022 Medicina publication, with the present analysis focusing specifically on biomechanical aspects of Achilles tendon moment arm alterations [21].

The aims of the present study are twofold: (1) to evaluate changes in the Achilles tendon moment arm after reconstruction using autologous semitendinosus and gracilis tendon grafts, based on pre- and postoperative radiographic measurements; (2) to assess the potential impact of moment arm alterations on the force-generating capacity of the triceps surae.

## 2. Materials and Methods

### 2.1. Study Design and Patient Characteristics

In the present study, a cohort of 25 patients (mean age: 44.5 years; age range: 20–64 years) who underwent Achilles tendon reconstruction using autologous semitendinosus and gracilis tendon grafts was analyzed [1]. The study group included 2 women and 23 men. The change in the Achilles tendon moment arm length was assessed by comparing the native insertion with the postoperative, reconstructed tendon insertion. In all analyses evaluating plantar flexor strength, the contralateral, non-operated lower limb was used as an internal reference, enabling objective assessment of functional and biomechanical outcomes.

The inclusion and exclusion criteria have been described in detail in a previous publication and are presented in Table 1. A total of 25 of the 30 consecutive patients initially enrolled at baseline were included in the analysis [21]. Five patients were excluded because the radiographs were of inadequate quality (e.g., incorrect projection or poor image resolution). No patients were excluded on the basis of clinical outcome, surgical complications, or treatment failure. All procedures were performed using the same surgical technique as described by Piontek et al. [12]. All patients followed a standardized rehabilitation protocol and were assessed according to the same evaluation scheme [22].

### 2.2. Surgical Procedure

Minimally invasive endoscopic reconstruction of chronic Achilles tendon ruptures, that was developed and described by our team in 2016, is performed using semitendinosus and gracilis tendon autografts with Endobutton fixation [11]. Under spinal anesthesia, with the patient in a prone position and a thigh tourniquet applied, two small endoscopic portals are created approximately 3 cm above the posterosuperior aspect of the calcaneus. These are used to remove adhesions and pathological tissue around the ruptured tendon. The hamstring grafts are harvested through a 3 cm incision over the proximal tibia, then cleaned, folded into a four-strand bundle, and prepared with an Endobutton loop. A midline incision just above the calcaneus is made to access the bone. Under fluoroscopic guidance, a tunnel is drilled through the calcaneus using a Kirschner wire. The distal end of the graft, fixed to the Endobutton, is inserted into this tunnel. The use of an Endobutton in this surgical technique necessitates anterior relocation of the Achilles tendon insertion on the calcaneus. The proximal ends of the graft are passed percutaneously through eight small skin incisions around the native Achilles tendon, tensioned, and tied at the most proximal incision site. All incisions are closed, a Jones dressing is applied, and the foot is placed in slight plantarflexion.

### 2.3. Rehabilitation Protocol

A specific rehabilitation protocol was developed and published by our team in conjunction with the aforementioned surgical technique [22]. It is divided into six stages, each with specific goals and therapeutic methods. In Stage I (weeks 0–2), the focus is on learning to walk with an orthosis, performing isometric and anti-thrombotic exercises, and encouraging independent home exercises. In Stage II (weeks 3–6), after the surgical wound has healed, water-based exercises are introduced, the operated limb is gradually loaded, ankle joint mobility is increased, and lower limb muscles are strengthened. Stage III (weeks 7–12) includes further improvement of range of motion, the introduction of resistance exercises, and gait pattern correction. In Stage IV (weeks 13–20), proprioception training, gait re-education, and more advanced strength and functional exercises are implemented. Stage V (weeks 21–32) focuses on correcting muscle imbalances, increasing strength and endurance, and gradually returning to sports-specific activities. In Stage VI (weeks 33–52), a detailed biomechanical and functional assessment is performed to determine the patient’s readiness to return to full sports activity.

### 2.4. Radiological Measurements

**Figure 1:**
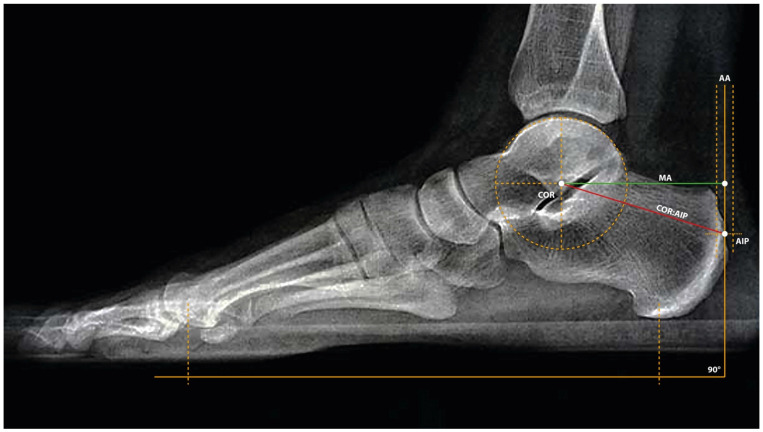
Lateral X-ray of the foot in a neutral position. The following were determined: COR—the center of rotation of the ankle joint; PP—the plantar plane as a line connecting the lower contour of the heel and the head of the first metatarsal bone; AA—the Achilles tendon axis as a line perpendicular to SP, passing through the center of the tendon; AIP—the tendon insertion point as the intersection of AA with the cortical outline of the calcaneal tubercle; MA (green line)—the lever arm as the distance from COR to the intersection of AA, perpendicular to SP passing through COR.

The measurement of the Achilles tendon moment arm length was based on lateral radiographic images of the entire foot, acquired in a standardized neutral position of the ankle joint 14 days after surgery in all patients in the study group. The following parameters were identified using routines custom-written in X-Twin X-ray system software v. 2.09.2152. Center of Rotation (COR): The center of ankle joint rotation was determined by manually overlaying a circle and aligning its curvature with the contour of the talar trochlea. The center of the fitted circle was defined as the ankle joint center of rotation [13]. Planar Plane (PP): The plantar plane was defined as a line passing through the lowest visible points of skin shadowing at (1) the inferior contour of the heel and (2) the projection of the first metatarsophalangeal joint. Achilles Axis (AA): The Achilles axis was determined by drawing a line perpendicular to the plantar plane, passing through the center of the Achilles tendon [23]. Achilles Insertion Point (AIP): For the native insertion (control group), the AIP was defined as the intersection between the AA line and the outer cortical contour of the calcaneal tuberosity. For the reconstructed tendon, the AIP was defined as the intersection between the AA line and the superior cortical contour of the calcaneal tuberosity. Distance of COR:AIP (red line): This was defined as the distance between the center of rotation of the ankle joint and the Achilles tendon insertion point. Achilles Tendon Moment Arm (MA, green line): The moment arm was determined by drawing a line perpendicular to the plantar plane passing through the center of rotation. The length of the moment arm (MA) was measured as the distance between the COR and the point where this perpendicular line intersected the Achilles axis. Due to the lack of contralateral comparative imaging and, naturally, the absence of pre-injury images, the determination of the Achilles insertion point can be approximated by identifying the most prominent of the two tubercles of the calcaneal tuberosity, located just above the inferior tubercle (which lies distal to the anatomical insertion). AIP is a point of force application by the tendon. At this point, the Achilles axis should be drawn perpendicular to the plantar plane, which approximates the anatomical direction of the Achilles tendon. All the lines and points described above are presented in Figure 1 below.

**Figure 2:**
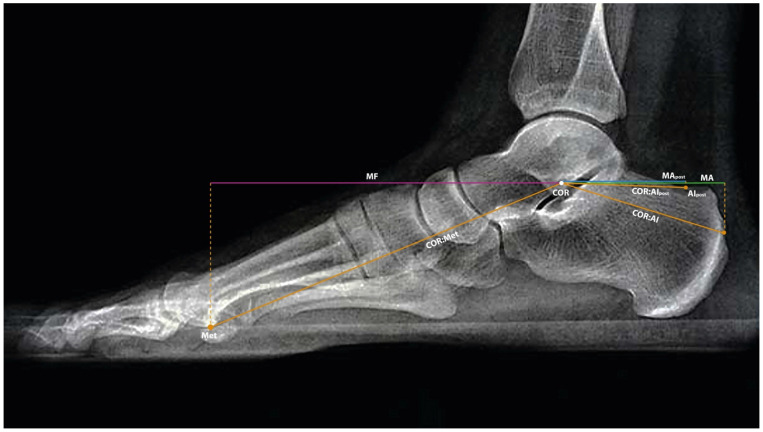
MA (green line)—anatomical lever arm of the Achilles tendon, hypothetical, corresponding to pre-injury conditions; MApost (blue line)—postoperative lever arm after tenodesis to the upper surface of the calcaneal tuberosity; MF (pink line)—lever arm of the foot, measured as the distance from the COR point to the head of the first metatarsal bone (Met), corresponding to the action of the plantar flexor muscles.

Three moment arms were considered for calculation and comparative analysis: Anatomical Achilles Moment Arm (MA, green line): a hypothetical, pre-injury Achilles tendon moment arm representing the anatomical condition prior to rupture; Postoperative Achilles Moment Arm (MApost, blue line): the measured Achilles tendon moment arm following surgical tenodesis to the superior surface of the calcaneal tuberosity; Foot Moment Arm (MF, pink line): the foot moment arm, defined as the distance through which the plantar flexor muscles exert pressure during plantarflexion, measured at the metatarsal head (Met) point. All the lines and points described above are presented in Figure 2 below [12,24,25].

All radiographic measurements were performed independently by two trained raters. Both raters were blinded to each other’s measurements and to the clinical outcomes. To assess measurement reliability, 10 randomly selected radiographs were re-evaluated after a 2-week interval. Inter- and intra-rater reliability for the primary outcome measure (Achilles tendon moment arm, MA) were quantified using a two-way random-effects intraclass correlation coefficient (ICC (2,1)) with 95% confidence intervals. The inter-rater ICC was 0.91 (95% CI: 0.82–0.96), while intra-rater ICC values were 0.94 and 0.92 for rater 1 and rater 2, respectively, indicating good to excellent agreement. The final value used in the analysis was the mean of the two measurements. The measurement protocol follows the method originally described by Deforth et al. [14], who reported an inter-rater ICC of 0.84 (95% CI: 0.73–0.91) and a test–retest reliability of ICC = 0.96 (95% CI: 0.93–0.98) on weight-bearing lateral radiographs.

### 2.5. Isometric Strength Assessment

Isometric strength of the plantar flexor muscles was assessed using the Biodex System 3 dynamometer [26]. The evaluation was conducted with participants in the prone position, secured by crossed stabilization belts across the back. The tip of the lateral malleolus was aligned with the rotational axis of the dynamometer [27]. The protocol consisted of three 5-second maximal voluntary isometric contractions (MVICs) of the plantar flexor muscles, each followed by a 5-second passive rest period, with the ankle joint maintained in a neutral position. Testing was performed first on the non-operated limb, followed by the operated limb [28,29].

### 2.6. Statistical Analysis

All calculations were performed using TIBCO Software Inc. (2017) Statistica (data analysis software system, version 13; https://www.statsoft.pl). The significance threshold for all analyses was set at *p* < 0.05. The data were described using descriptive statistics, including mean, standard deviation (SD), minimum and maximum values, as well as counts (*n*) and percentages (%). The Shapiro–Wilk test was used to assess the normality of the distribution for each variable. The paired Student’s *t*-test was used to compare the moment arm length of the Achilles tendon, taking into account the arm length determined from the native tendon insertion, considered as the preoperative length, and the postoperative arm length determined using X-ray imaging. The difference in plantar flexor strength between the operated and non-operated limbs before and after the procedure was evaluated using the Wilcoxon signed-rank test (*Z*). Changes in plantar flexor muscle strength (PF [N]) over time (preoperative—12 months; postoperative—24 months) and between limbs (operated vs. non-operated) were analyzed using a two-way repeated measures analysis of variance (2 × 2 ANOVA). Effect sizes (*d*) were calculated as the mean difference divided by the standard deviation of the difference and categorized as follows according to Cohen’s criteria: small (≥0.2 and <0.5), moderate (≥0.5 and <0.8), and large (≥0.8). To identify patients who achieved a clinically meaningful change, the Minimal Clinically Important Difference (MCID) thresholds were used, calculated as 0.2 × SD for a small change and 0.5 × SD for a substantial change based on the baseline value of the variable. For Achilles tendon moment arm length in the operated limb, the MCID thresholds were 1 mm (small change) and 3 mm (substantial change). For plantar flexor muscle strength, the thresholds were 112 N (small change) and 281 N (substantial change) for the operated leg and 104 N and 261 N for the non-operated leg, respectively. The relationship between the degree of moment arm shortening and plantar flexor strength in the operated limb before and after surgery was assessed using Spearman’s rank correlation. Correlation coefficients were compared using Fisher’s *Z*-test for dependent correlations. The magnitude of differences between correlations was further evaluated using Cohen’s *q*, with the following interpretation: *q* < 0.1—no effect; 0.1 ≤ *q* < 0.3—small effect; 0.3 ≤ *q* < 0.5—moderate effect; *q* ≥ 0.5—large effect [30].

## 3. Results

### 3.1. Achilles Tendon Moment Arm Length

Based on data collected from 25 patients who underwent surgical reconstruction of the Achilles tendon, the length of the Achilles tendon moment arm in the operated limb was analyzed by comparing the native insertion with the postoperative, reconstructed tendon insertion.

The analysis revealed a statistically significant shortening of the Achilles tendon moment arm length in the operated limb following surgery (*t* = 16.95, *p* < 0.05). The mean difference between the two measurement points was 6.6 mm, with a large effect size (Cohen’s *d* = 1.20).

Within the study group, the smallest observed shortening was 4.0 mm, while the greatest was 10.6 mm (Table 2). All patients (*n* = 25) exceeded the threshold for the MCID, which was set at ≥3 mm.

### 3.2. Plantar Flexor Muscle Strength

Plantar flexor muscle strength was compared between the operated and non-operated lower limbs at two time points: 12 and 24 months after surgery. The analysis revealed a trend toward statistical significance for the interaction effect between limb and time (*p* < 0.05).

**Figure 3:**
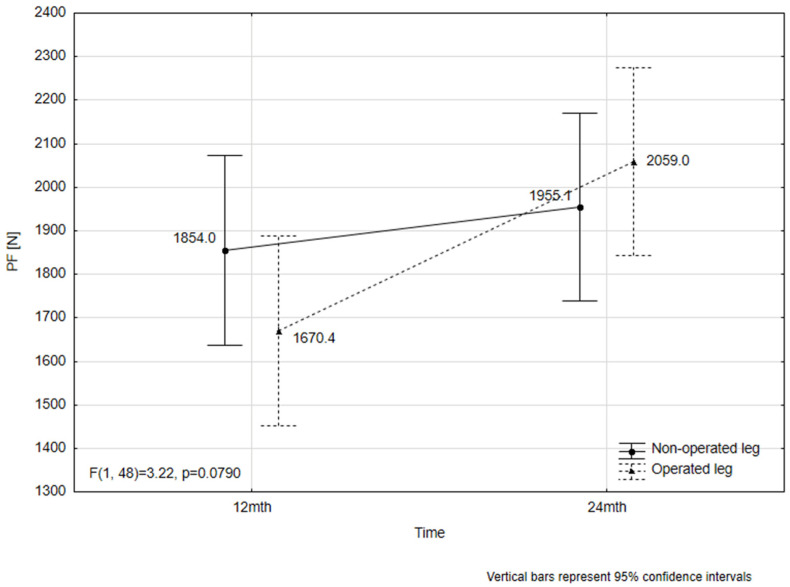
Plantar flexor muscle force (based on torque and moment arm) 12 and 24 months after surgery.

In the operated limb, plantar flexor strength increased by an average of 388.6 N at 24 months postoperatively compared to 12 months, a statistically significant improvement (*p* = 0.0067) with a moderate effect size (Cohen’s *d* = 0.71). In contrast, the change observed in the non-operated limb was 101.1 N, which was not statistically significant (*p* = 0.8098) (Figure 3).

Among patients who underwent surgery, 48% (*n* = 12) reached the threshold for a substantial clinically meaningful improvement (≥281 N), while an additional 20% (*n* = 5) achieved a small but clinically relevant improvement (≥112 N).

Furthermore, the difference in plantar flexor strength 12 months and 24 months after surgery was compared between limbs. The improvement in the operated limb (287.5 N) was significantly greater than in the non-operated limb, as confirmed by the Wilcoxon signed-rank test (*Z* = 3.83, *p* = 0.0001).

### 3.3. Strength vs. Moment Arm Shortening

No statistically significant relationship was found between the degree of Achilles tendon moment arm shortening and the recovery of plantar flexor muscles strength in the operated limb 12 months postoperatively (*r* = −0.03, *p* = 0.8695) or at 24 months (*r* = −0.18, *p* = 0.3880).

The correlations between the extent of moment arm shortening and plantar flexor muscles strength 12 and 24 months after surgery did not differ significantly (*Z* = 0.69, *p* = 0.246), and the effect size of the correlation difference was negligible (Cohen’s *q* = 0.01).

## 4. Discussion

The results of this study confirm that reconstruction of chronic Achilles tendon ruptures using autologous semitendinosus and gracilis tendon grafts leads to a significant shortening of the plantar flexor moment arm. The mean shortening was 6.6 mm, corresponding to approximately 11.5% of the estimated pre-injury value, and exceeded the MCID in all patients. From a biomechanical perspective of the ankle joint, such a change may potentially limit the triceps surae muscle’s ability to generate plantarflexion torque, as reported by Baxter and Piazza [12,24].

It was initially hypothesized that shortening the Achilles tendon moment arm would negatively influence the restoration of triceps surae muscle strength. However, the obtained results do not indicate a statistically significant difference. Despite this theoretical risk, analysis of muscle strength parameters revealed no significant correlation between the degree of moment arm shortening and plantar flexor strength at either 12 or 24 months postoperatively. Moreover, two years after surgery, the operated limb did not differ significantly in strength from the contralateral limb, and in some cases, it was even stronger. A previous publication on isokinetic testing demonstrated that 12 and 24 months after Achilles tendon reconstruction with hamstring autografts, no significant differences in the peak torque angle of the plantar flexors were observed between the operated and contralateral sides in this cohort [21]. These findings suggest that a long-term, progressive rehabilitation program can fully compensate for moment arm shortening, consistent with the conclusions of Zatsiorsky and Prilutsky [31] and Wren et al. [32], who highlighted the ability of the muscle–tendon unit to adapt under appropriate training conditions [27,33].

Possible mechanisms underlying this compensation include collagen remodeling within the graft over time, changes in its elastic properties in response to load, adaptive adjustments in motor unit recruitment strategies and increased activation of other plantar flexor muscles (tibialis posterior, flexor digitorum longus, flexor hallucis longus, peroneus longus and brevis) [18]. Unfortunately, it was not possible to assess compensatory movements from other muscle groups using the Biodex system, as this equipment operates exclusively in a single plane—here, specifically, it operates in the sagittal plane. Consequently, it was not feasible to determine whether the patient exhibited eversion or inversion during the testing procedure.

It should be noted, however, that despite the significant mean increase in strength (by 388.6 N at 24 months), nearly half of the patients did not reach the threshold for substantial improvement (≥281 N), suggesting that in some individuals, the shortened moment arm may still limit maximal strength potential, even if not detectable in simple correlation analyses [34]. When these data are considered alongside patient-reported outcome scores and objective functional evaluations collected in this cohort, the current results are consistent with previously reported observations [21]. A significant improvement at 24 months postoperatively compared with 12 months in the Achilles Tendon Rupture Score (ATRS), the EQ-5D-5L questionnaire, and patient-reported pain and satisfaction has previously been reported. A similar trend was observed in the battery of functional tests, including the weight-bearing lunge test, heel-rise test, single-hop test, and both isometric and isokinetic strength measurements of the ankle plantar flexors and dorsiflexors [21]. Considering the above findings, patients should be informed prior to surgery that rehabilitation may extend for approximately two years, or at a minimum until comprehensive functional assessments—including strength and dynamic performance testing—confirm adequate recovery. Moreover, the impact of resuming normal daily activities and physical exercise beyond the 12-month postoperative period warrants further investigation. It is common for patients to discontinue structured physiotherapy after this time, although functional performance continues to improve. This highlights the need for additional studies and a more in-depth analysis of patient behavior during the 12–24-month recovery interval [34].

From a surgical standpoint, the applied technique involves anchoring the graft to the superior surface of the calcaneal tuberosity, inevitably shifting the insertion point away from its anatomical location and causing moment arm shortening [11]. Alternative approaches proposed in the literature include more anatomically positioned reconstructions [35,36], the use of longer allograft or synthetic grafts [32], or bone anchors placed at the native insertion site [37]. Comparative studies with parallel groups are required to assess their effectiveness.

A further consideration relates to the relatively small cohort size and the limited number of female participants in the study. Reconstruction of chronic Achilles tendon ruptures with hamstring autografts is not a first-line procedure. It is typically performed as a secondary intervention after failed primary treatment or in cases of neglected ruptures. Consequently, the pool of eligible patients is inherently restricted, which limits the feasible sample size in clinical studies of this type. In addition, several patients initially considered for inclusion were excluded due to inadequate radiographic quality (e.g., incorrect projection or poor image resolution), further reducing the final study group. These factors partly explain the modest number of participants and the imbalance in sex distribution. Although we did not observe sex-specific trends in the measured biomechanical or functional outcomes, we acknowledge that the small number of female patients reduces the statistical power to detect potential sex-related differences. Future multicenter studies with larger, more gender-balanced cohorts would be valuable for confirming the generalizability of our findings.

Several limitations of the present study should be acknowledged: the lack of actual preoperative moment arm measurements (values were estimated from the postoperative X rays), simplifications in defining the Achilles tendon axis on radiographs, difficulty in replicating the curvature of the talar trochlea, small sample size, and the absence of a control group. Although inter- and intra-rater reliability demonstrated good to excellent agreement, repeated measurements were performed only in a subset of radiographs, and minor measurement error inherent to two-dimensional radiographic assessment cannot be completely excluded. The lack of preoperative plantar flexor strength measurements precludes assessment of absolute pre-to-post treatment changes. However, the contralateral, non-operated limb was used as an internal benchmark, which allows for meaningful interpretation of the results. Additionally, the measurements were obtained under static conditions, which do not fully reflect the dynamic changes in the moment arm during movement. This topic should be addressed in future gait analysis or 3D kinematic studies.

## 5. Conclusions

Although reconstruction of chronic Achilles tendon ruptures with this technique inevitably results in moment arm shortening, a well-structured, long-term, and progressive rehabilitation program can largely offset the potential adverse biomechanical effects. Patients should be informed that returning to full function may require up to two years of systematic effort. In some cases, residual strength limitations may persist despite the absence of measurable deficits in standard isometric tests.

## Figures and Tables

**Table 1:** Inclusion and exclusion criteria of the study group.

Inclusion Criteria	Exclusion Criteria
>18 years of age	Contraindications to MRI (ferromagnetic implants, prostheses, implants)
<65 years of age	Active infectious process
	Metabolic diseases that prevent surgical treatment
Achilles tendon damage: chronic rupture of the Achilles tendon (>6 weeks) with stump retraction of more than 3 cm chronic partial damage to the Achilles tendon (damage covering more than 50% of the tendon fibers) with its dysfunction Previous failure of conservative or surgical treatment	Achilles tendon damage: as determined by the operating surgeon: acute Achilles tendon rupture with stump retraction < 3 cm first-time Achilles tendon rupture

**Table 2:** Moment arm lengths.

	Preoperative MA (Estimated *)	Postoperative MA (MApost **)	*p* Value (Preoperative vs. Postoperative)	Mean Shortening
Mean (mm)	57.5	50.9	<0.0001	6.6
SD (mm)	5.38	5.59		1.96
Max (mm)	69.1	63.5		10.6
Min (mm)	47.4	41.4		4.0

Values are presented as maximum, minimum, mean ± standard deviation. The given values are *p*; *p*-value irrelevant. * Anatomical lever arm of the Achilles tendon. ** Postoperative Achilles Moment Arm.

## Data Availability

Data are available from the corresponding author upon reasonable request.
